# Feasibility and acceptability of an online psychological group intervention for allogeneic hematopoietic stem cell transplantation inpatients

**DOI:** 10.1002/hem3.70237

**Published:** 2025-10-13

**Authors:** Karl Haller, Asita Behzadi, Johannes C. Ehrenthal, Lars Bullinger, Johann Ahn

**Affiliations:** ^1^ Department of Hematology, Oncology, and Cancer Immunology Charité‐Universitätsmedizin Berlin Berlin Germany; ^2^ Department of Psychology University of Cologne Cologne Germany; ^3^ National Center for Tumor Diseases (NCT) Berlin Berlin Germany; ^4^ German Cancer Consortium (DKTK) Berlin Berlin Germany

Allogeneic hematopoietic stem cell transplantation (allo‐HSCT) is the only curative option for various hematological malignancies and autoimmune diseases, and typically requires a 4–6‐week hospital stay in protective isolation.^1^ Patients frequently endure intense physical and psychological distress, with up to a third developing clinically significant anxiety or depression.^2^ Isolation limits contact with family and peers, exacerbating distress and thus poses a particular challenge.^3^ Despite this, few supportive interventions target allo‐HSCT patients during hospitalization, and none focus on group therapy in this phase.^4^ Yet group programs can benefit cancer patients by fostering peer support, coping strategy exchange, and emotional self‐efficacy.^5^, ^6^

Challenges such as immunosuppression, unpredictable treatment schedules, high symptom burden, and potential emotional strain when sharing concerns with others complicate group interventions. A recent prehabilitation group program for allo‐HSCT patients reported similar obstacles, including time constraints, other medical priorities, and low initial distress to motivate participation.^7^ Given these complexities, feasibility studies are necessary before larger trials. This study developed and tested a novel online group intervention tailored to allo‐HSCT inpatients.

Following CONSORT guidelines for pilot and feasibility trials, a quasi‐experimental matched‐control study was conducted to assess the feasibility, acceptability, and exploratory outcomes. Feasibility was assessed via recruitment and retention rates, while acceptability was evaluated using self‐report satisfaction measures. Potential adverse effects and deviations from the intervention manual were monitored. A randomized controlled design was deemed inappropriate due to the early stage of intervention development and high disease burden, instead, a matched‐control design allowed all interested patients to participate while providing comparative data. The target sample size was 18 per group, considered realistic for the 6‐month study period. Eligibility criteria included age > 18, current inpatient allo‐HSCT, and sufficient German language proficiency. Assessments occurred at admission and discharge.

The intervention was developed and manualized based on literature, patient interviews, and expert input, including hematologists, nurses, psycho‐oncologists, and patient representatives.^8^ Patients expressed interest in peer exchange, flexible participation, and psycho‐oncological guidance. The final format consisted of weekly 60‐min sessions via a privacy‐compliant video platform, featuring tailored group rules, an opening round, thematic discussions (topics: 1. Coping with Isolation and Daily Routines, 2. Communication and social support, 3. Coping with illness and symptoms, and 4. Discharge from hospital and return to everyday life, detailed description of the topics can be found in the supplement), optional relaxation exercises, and accommodations for medical interruptions. Devices were provided to patients as needed. Controls were enrolled before the intervention was available and received treatment as usual, including individual psycho‐oncological counseling if requested.

Outcome measures:

- ISOLA scale: Measures isolation‐related suffering (1–5 scale), reliability *α* = 0.89.^9^, ^10^
- Patient Health Questionnaire‐4 (PHQ‐4): Assesses depression and anxiety symptoms (0–12 scale), *α* = 0.82, cutoff ≥ 6 indicates clinical distress.^11^
- Distress thermometer: Visual analog scale 0–10, ≥5 indicates significant distress.^12^
- Functional Assessment of Cancer Therapy‐General (FACT‐G): Quality of life across physical, social, emotional, and functional well‐being (0–108), higher is better.^13^
- Stanford emotional self‐efficacy scale‐cancer (SESES‐C): Emotional self‐efficacy scale (0–100), *α* = 0.84 in German translation.^14^
- Group Cohesiveness Scale (GCS): Group Cohesion Scale (1–5 Likert), *α* = 0.90 in German translation.^15^

Analyses were conducted using IBM SPSS 29, based on the intention‐to‐treat principle. Missing data for one deceased patient were imputed. One patient not attending any session was included in the feasibility but excluded from the effect analyses. Chi‐square and *t*‐tests examined demographic differences; repeated‐measures analysis of variance (ANOVA) tested outcomes (with effect sizes as *η*²); significance set at P < 0.05 with Holm–Bonferroni correction; distress score and PHQ‐4 score Johnson‐transformed due to a lack of normal distribution. Non‐transformed mean values are reported for better comparability.

Out of 57 patients admitted, 39 met the inclusion criteria; 18 agreed to participate and were enrolled in the intervention (see Supplement S1). There were no statistically significant baseline differences in demographics or clinical variables between the control and intervention groups (Table 1).

**Table 1:** Participant's sociodemographic and medical characteristics.

	IG (*n* = 17)	CG (*n* = 19)	
	*n* (%)/*M* (SD)	*n* (%)/*M* (SD)	*t*‐Test/chi^2^
Gender			0.738
Male	9 (53%)	9 (47%)	
Female	8 (47%)	10 (53%)	
Age (mean, SD)	52.2 (13.6)	53.4 (14.1)	0.798
Educational level			
Academics	12 (71%)	8 (42%)	0.086
Current occupation			0.484
Employed	8 (47%)	9 (47%)	
Self‐employed	3 (18%)	3 (16%)	
Unemployed	0 (0%)	3 (16%)	
Retired	5 (29%)	3 (16%)	
In training	1 (6%)	1 (5%)	
Mother tongue			0.593
German	15 (88%)	18 (95%)	
Other	2 (12%)	1 (5%)	
Partnership			0.858
Currently in partnership	13 (77%)	15 (79%)	
Children			0.410
0	4 (23%)	9 (47%)	
1	4 (23%)	3 (16%)	
≥2	9 (53%)	7 (37%)	
Monthly income	10 (59%)	7 (37%)	0.187
≥3000 euro/month			
Primary disease			0.350
Primary AML (including MPAL)	12 (71%)	10 (53%)	
MDS/MPN/overlap	1 (6%)	5 (26%)	
Lymphatic disorders	2 (12%)	3 (16%)	
Bone marrow failure/special cases	2 (12%)	1 (5%)	
Disease status			0.296
Primary diagnosis	13 (76%)	17 (89%)	
Relapse	4 (24%)	2 (11%)	
Number of allo‐HSCT			0.056
First	14 (82%)	19 (100%)	
Second	3 (18%)	0 (0%)	
Type of allo‐HSCT			0.326
Haploident	3 (18%)	1 (5%)	
HLA identical	14 (82%)	18 (95%)	
Fever			0.650
Afebrile	3 (18%)	2 (11%)	
Febrile	14 (82%)	17 (89%)	
Oral mucositis			0.813
Grade 0	5 (30%)	6 (32%)	
Grade 1	1 (6%)	3 (16%)	
Grade 2	5 (29%)	6 (32%)	
Grade 3	5 (29%)	3 (16%)	
Grade 4	1 (6%)	1 (5%)	
Acute graft versus host disease			0.518
Grade 0	12 (70%)	11 (58%)	
Grade 1	3 (18%)	6 (32%)	
Grade 2	2 (12%)	1 (5%)	
Grade 3	0	0 (0%)	
Grade 4	0	1 (5%)	
Length of stay on the transplant unit	37.41 (12.96)	43.00 (9.64)	0.074
Distress on admission to hospital (mean, SD)	4.18 (2.24)	4.16 (2.65)	0.982
Depression/anxiety on admission to hospital (mean, SD)	3.82 (2.77)	3.58 (3.15)	0.807

Abbreviations: allo‐HSCT, allogeneic hematopoietic stem cell transplantation; AML, acute myeloid leukemia; CG, control group; HLA, human leukocyte antigen; IG, intervention group; MDS, myelodysplastic syndrome; MPAL, mixed‐phenotype acute leukemia; MPN, myeloproliferative neoplasm; *t*‐test/chi^2^, P value for Fisher's exact test, Pearson chi‐square test, and *t*‐test for independent samples.

Sixteen group sessions were scheduled; 12 were conducted with at least two participants. On average, five candidates were identified per session (range 3–7), with three attending (range 2–4). Barriers included medical appointments (36%), symptom burden (32%), visits (16%), intensive care unit (ICU) transfers (12%), and technical issues (4%). Attendance rates: 94% attended ≥1 session, 72% ≥2, 44% ≥3, and 5% all four. One session was shortened due to discomfort; one patient discontinued due to distress from group discussions.

Most participants found topics meaningful (87%) and session length appropriate (80%). In total, 47% felt the number of sessions was sufficient; 73% desired additional sessions. Therapist facilitation was rated positively (93%), and 93% would recommend the intervention. Group cohesion averaged *M* = 3.82 (SD = 0.78).

At discharge, 37% of controls versus 18% of intervention patients screened positive for clinical distress (PHQ‐4 ≥ 6), highlighting clinical relevance. Significant group × time interaction for depression/anxiety symptoms (PHQ‐4), with reduction in intervention group and increase in controls (*F*(1,35) = 6.68, P = 0.009, *η*² = 0.183). No significant differences observed for distress (P = 0.905), quality of life (P = 0.495), emotional self‐efficacy (P = 0.917), or isolation (P = 0.450) (Table 2).

**Table 2:** Means, standard deviations, and analysis of variance (ANOVA) results for outcome measures by group and time point.

	Pre (T0)		Post (T1)		
	IG (*n* = 17)	CG (*n* = 19)	IG (*n* = 17)	CG (*n* = 19)	Intervention effect			
	*M* (SD)	*M* (SD)	*M* (SD)	*M* (SD)	df	*F*	P	Part. *η*²
Distress	4.18 (2.24)	4.16 (2.65)	4.18 (2.19)	4.74 (3.31)	1, 35	0.014	0.905	0.000^a^
Depression/anxiety	3.82 (2.77)	3.58 (3.15)	3.01 (2.86)	5.05 (3.52)	1, 35	6.68	0.009*	0.183^a^
Quality of life	72.94 (13.92)	75.46 (16.33)	68.34 (18.14)	74.16 (15.73)	1, 35	0.473	0.496	0.014
Emotional self‐efficacy	76.19 (12.24)	74.18 (14.81)	80.52 (9.74)	78.14 (13.96)	1, 35	0.011	0.917	0.000
Suffering from isolation	3.04 (0.80)	2.88 (0.84)	3.18 (0.86)	2.82 (0.86)	1, 35	0.583	0.450	0.017

Abbreviations: CG, control group; df, degrees of freedom; *F*, *F*‐value; IG, intervention group; *M*, mean; P, probability value; part. *η*², partial eta squared; SD, standard deviation.

^a^ The data were Johnson‐transformed due to a lack of normal distribution. *M* and SD are shown untransformed for better comparability.

* P < 0.05 (adjusted using the Holm–Bonferroni correction).

The results suggest that a psycho‐oncological group intervention for patients undergoing allo‐HSCT is both feasible and well accepted, a first in this population. The digital format was well‐managed by patients, and nearly half of the eligible patients enrolled, comparable to prior studies.^7^ While only 5% attended all four sessions, 72% attended at least two and 44% at least three, likely reflecting somatic symptoms, side effects, and medical appointments rather than low acceptability (73% expressed interest in additional sessions), and although the average inpatient stay (approx. 5 weeks) limited full attendance for some, the intervention still fostered a sense of connection. Group cohesion scores were consistent with those of other, even significantly longer group therapy programs.^16^ The shared treatment experience created a foundation for mutual support despite heterogeneity. Differences within the group were constructively utilized; for example, patients nearing discharge shared positive experiences that reassured others. From a psychological perspective, even single sessions may provide relief, while more sustained effects generally require repeated exposure.

Our preliminary observations suggest the intervention may protect against worsening depression and anxiety. The pattern of symptom progression in the control group aligns with findings from similar studies on psychopathology in allo‐HSCT patients^2^; however, given the small sample size, these findings should be interpreted with caution. The absence of effects on distress, quality of life, isolation, or emotional self‐efficacy may relate to the limited intervention dose (median two sessions) and the overwhelming physical/medical burden during transplantation. Longer or more intensive interventions may be needed for these broader psychosocial outcomes.

The group intervention did not reduce demand for individual psycho‐oncological support, suggesting complementary roles; similar proportions of patients in the intervention (35.3%) and control groups (36.8%) requested individual sessions, with comparable frequency (2.0 vs. 2.7) and duration (96 vs. 111 min).

Limitations include small sample size, lack of randomization, possible self‐selection bias as well as imbalances in socioeconomic status and prior transplant experience between the groups. Although these imbalances were not statistically significant, this may reflect the limited sample size rather than the absence of meaningful differences. Socioeconomic factors could have facilitated engagement and adherence (e.g., through higher health literacy, greater psychosocial resources, or more flexibility), while patients undergoing a second allo‐HSCT may have had a greater psychological burden or influenced group dynamics. Such factors could therefore have contributed to differences in the outcome measures and should be further examined in future studies; a randomized design would help minimize this risk. Nonetheless, the intervention was safe, with therapeutic leadership effectively managing distress from difficult group content and suggesting that even minimal participation may help counteract the typical increase in depressive symptoms during allo‐HSCT. Future research should explore larger randomized trials, optimal group composition, session frequency, and post‐discharge follow‐ups.

This pilot study confirms the feasibility and acceptability of an online psycho‐oncological group intervention for allo‐HSCT inpatients and shows preliminary protection against depression and anxiety. The approach meets the specific needs of this population in a vulnerable situation and appears resource‐efficient. To strengthen the evidence base, assess long‐term outcomes, and inform optimal implementation, larger trials are needed. Similar formats might benefit other patients in protective isolation, such as those undergoing autologous transplantation or induction therapy.

## AUTHOR CONTRIBUTIONS

**Karl Haller**: Conceptualization; data curation; formal analysis; investigation; writing—original draft; writing—review and editing; visualization; funding acquisition; validation; methodology. **Asita Behzadi**: Conceptualization; methodology; data curation; investigation; validation; writing—review and editing. **Johannes C. Ehrenthal**: Conceptualization; methodology; formal analysis; validation; writing—review and editing. **Lars Bullinger**: Writing—review and editing. **Johann Ahn**: Writing—review and editing; conceptualization; methodology; supervision; resources.

## CONFLICT OF INTEREST STATEMENT

The authors declare no conflicts of interest.

## ETHICS STATEMENT

Approved by the Ethics Committee of Charité – Universitätsmedizin Berlin (EA1/072/24).

## FUNDING

This research was funded by the foundation Stefan‐Morsch‐Stiftung. Open Access funding enabled and organized by Projekt DEAL.

## PATIENT CONSENT STATEMENT

All participants provided written informed consent, in accordance with the Declaration of Helsinki and GCP guidelines.

## Supporting information

Supporting Information.

## Data Availability

The data that support the findings of this study are available from the corresponding author upon reasonable request.
