# Supplementary material for: Feasibility and acceptability of an online psychological group intervention for allogeneic hematopoietic stem cell transplantation inpatients
Source: Hemasphere. 2025 Oct 13;9(10):e70237. doi: 10.1002/hem3.70237 (PMC12516907; doi:10.1002/hem3.70237)
Supplement: Supplementary file 1 — Supporting Information. [file HEM3-9-e70237-s001.docx]

**Table (Supplement).** Overview of the content of each group session

| **Session # and Topic** | **Description** |
| --- | --- |
| 1. Coping with Isolation and Daily Routines | Focuses on managing the psychological strain of protective isolation, maintaining daily routines, and addressing fears such as loneliness, infection, and sleep disturbances. Group discussions aim to enhance coping skills and mutual learning. |
| 2. Communication and social support | Explores communication strategies with family, friends, and healthcare providers, emphasizing boundaries, clear expectations, and reducing misunderstandings. Participants reflect on their own communication habits and strengthen social support networks. |
| 3. Coping with illness and symptoms | Addresses challenges related to treatment side effects, existential fears, and disease processing. Group reflections and shared strategies aim to improve quality of life and promote adaptive coping mechanisms. |
| 4. Discharge from hospital and return to everyday life | Focuses on discharge planning, managing immunosuppression, and rebuilding daily routines. Participants explore strategies to regain confidence and address fears, such as relapse anxiety, during the return to normal life. |
